# *Plasmodium falciparum *glutamate dehydrogenase a is dispensable and not a drug target during erythrocytic development

**DOI:** 10.1186/1475-2875-10-193

**Published:** 2011-07-14

**Authors:** Janet Storm, Jan Perner, Isabela Aparicio, Eva-Maria Patzewitz, Kellen Olszewski, Manuel Llinas, Paul C Engel, Sylke Müller

**Affiliations:** 1 Institute of Infection, Immunity & Inflammation, Wellcome Trust Centre for Molecular Parasitology, College of Medical, Veterinary and Life Sciences, Sir Graeme Davies Building, University of Glasgow, 120 University Place, Glasgow G12 8TA, UK; 2 University College Dublin, School of Biomolecular and Biomedical Sciences, Conway Institute, University College Dublin, Belfield, Dublin, Republic of Ireland; 3 Department of Molecular Biology, Lewis-Sigler Institute for Integrative Genomics, Princeton University, Princeton, NJ 08544, USA; 4 Current address: Institute of Genetics, QMC, University of Nottingham, Nottingham NG7 2UH, UK

## Abstract

**Background:**

*Plasmodium falciparum *contains three genes encoding potential glutamate dehydrogenases. The protein encoded by *gdha *has previously been biochemically and structurally characterized. It was suggested that it is important for the supply of reducing equivalents during intra-erythrocytic development of *Plasmodium *and, therefore, a suitable drug target.

**Methods:**

The gene encoding the NADP(H)-dependent GDHa has been disrupted by reverse genetics in *P. falciparum *and the effect on the antioxidant and metabolic capacities of the resulting mutant parasites was investigated.

**Results:**

No growth defect under low and elevated oxygen tension, no up- or down-regulation of a number of antioxidant and NADP(H)-generating proteins or mRNAs and no increased levels of GSH were detected in the D10^Δ*gdha *^parasite lines. Further, the fate of the carbon skeleton of [^13^C] labelled glutamine was assessed by metabolomic studies, revealing no differences in the labelling of α-ketoglutarate and other TCA pathway intermediates between wild type and mutant parasites.

**Conclusions:**

First, the data support the conclusion that D10^Δ*gdha *^parasites are not experiencing enhanced oxidative stress and that GDHa function may not be the provision of NADP(H) for reductive reactions. Second, the results imply that the cytosolic, NADP(H)-dependent GDHa protein is not involved in the oxidative deamination of glutamate but that the protein may play a role in ammonia assimilation as has been described for other NADP(H)-dependent GDH from plants and fungi. The lack of an obvious phenotype in the absence of GDHa may point to a regulatory role of the protein providing glutamate (as nitrogen storage molecule) in situations where the parasites experience a limiting supply of carbon sources and, therefore, under *in vitro *conditions the enzyme is unlikely to be of significant importance. The data imply that the protein is not a suitable target for future drug development against intra-erythrocytic parasite development.

## Background

Glutamate dehydrogenases (GDH) are enzymes that catalyse the reversible oxidative deamination of L-glutamate to form α-ketoglutarate and ammonia using NADP(H) or NAD(H) as co-factors:

The enzymes are at the branch point of nitrogen and carbon metabolism and either assimilate ammonia and provide glutamate as nitrogen storage molecule or dissimilate ammonia and provide α-ketoglutarate that can feed into tricarboxylic acid (TCA) metabolism. There are three different types of GDH depending on their co-factor specificity. The enzymes specific for NAD(H) generally catalyse the oxidative deamination of L-glutamate (to generate α-ketoglutarate) with an alkaline pH optimum; the enzymes specific for NADP(H) usually carry out the reductive amination of α-ketoglutarate (to generate L-glutamate) with a neutral pH optimum. The third type, represented by the vertebrate GDH enzymes, can use both co-factors for the deamination of L-glutamate [1].

The human malaria parasite *Plasmodium falciparum *contains three genes encoding potential GDH proteins; two are found on chromosome 14 (PF14_0164 and PF14_0286, encoding GDHa and b, respectively) and one is present on chromosome 8 (PF08_0132 encoding GDHc) [2,3]. GDHa and GDHb are NADP(H)-dependent and the primary sequence of GDHb suggests the protein is located in the apicoplast, while the localisation and co-factor specificity is not predictable for GDHc. This presence of multiple GDH proteins is reminiscent of plants and fungi [4-7]. In plants, the two NAD(H)-dependent GDHs are essential for carbon metabolism while the role of the putative NADP(H)-dependent GDH is thought to provide glutamate through NH_4_^+ ^assimilation [7-10]. In fungi, the NADP(H) dependent GDH are also involved in NH_4_^+ ^assimilation [4,5,11].

*Plasmodium falciparum gdha *was previously cloned and recombinant protein was biochemically and structurally characterized [3,12-14]. The kinetic parameters are comparable to other GDH enzymes using NADP(H) as co-factor and suggest that the enzyme catalyses both the oxidative deamination of glutamate and the reductive amination of α-ketoglutarate [13]. It was hypothesized that one of the major roles of GDHa is to provide reducing equivalents in form of NADPH that is required for downstream redox reactions, carried out by the disulphide oxidoreductases glutathione reductase and thioredoxin reductase [14-17]. Thus, the enzyme was considered integral to the parasite's antioxidant machinery and thought to be a potential drug target [3,13,14,18,19]. Recently, it was shown that *P. falciparum *growth was susceptible to inhibition by the GDH inhibitor, isophthalic acid. However, this effect was only achieved at high μM concentrations suggesting that the inhibitory effect may also be attributable to inhibition of one or both of the other two GDH proteins [3]. A recent high-throughput drug screen against several potential drug targets for the development of *Plasmodium*, which included GDHa, did not identify any specific compounds that were inhibitory for the enzyme, questioning the suitability of GDHa as a drug target because it may simply not be "druggable" [20].

To genetically validate GDHa as a potential target for the design of new anti-malarials during their intra-erythrocytic development this study generated *gdha *null mutants of *P. falciparum *and investigated their phenotypes with respect to their abilities to withstand oxidative challenges, their expression levels of antioxidant proteins and the metabolic fate of isotope labelled glutamine.

## Methods

### Materials

WR99210 was a kind gift from Jacobus Pharmaceuticals (USA). Plasmids pHH1 and pHH2 were kind gifts from Professor A. F. Cowman (The Walter and Eliza Hall Institute, Melbourne, Australia) [21,22]. Human blood was obtained from the Glasgow blood transfusion services. Specific antibodies, used in western blot experiments, were generated in rabbit or rat using recombinantly expressed protein [23-28] (Eurogentec, Belgium). Secondary antibodies were obtained from Promega. Anti-GST antibodies were a kind gift from Professor E. Liebau (University of Münster, Germany), anti-SOD 1 and 2 were obtained from Dr J. Khalife (University of Lille Nord de France, Institut Pasteur de Lille, France) and GDHa antibodies were a gift from Professor L. Krauth-Siegel (University of Heidelberg, Germany). [8-^3^H]-hypoxanthine (specific activity: 10-30 Ci/mmol) was from American Radiolabeled Chemicals. U-[^13^C] glutamine and U-[^13^C-^15^N] glutamine were purchased from CK gas, UK. All other chemicals were purchased from Sigma UK unless otherwise stated.

### Knockout and expression constructs of *P. falciparum gdha*

The knockout and expression fragments of the *P. falciparum gdha *gene were amplified from *P. falciparum *3D7 or D10 genomic DNA using Pfx Supermix (Invitrogen, UK). The specific oligonucleotide primers 5'-GCGC**AGATCT**GGTTTACGATTTCATCC-3' (sense) and 5'-CGCG**CTCGAG**(TTA)TTGCTGCTTTTGATGG-3' (antisense) with the BglII and XhoI restriction sites, respectively, in bold, and an artificial stop codon (in brackets) within the antisense oligonucleotide, were used to generate the 841 bp insert equivalent to nucleotides 339-1179 of the *gdha *open reading frame for the *gdha *knockout construct. For the *gdha-GFP *construct the primers 5'-GCGC**AGATCT**(ATG)AGTGCTCTTAAAG-3' (sense) and 5'-CGCG**CCTAGG**AAAACAACCTTG-3' (anti sense) with the BglII and AvrII restriction sites, respectively, in bold, and an artificial start codon (in brackets) within the sense oligonucleotide were used to generate a full-length protein with a C-terminal GFP-tag. The PCR products were subcloned into the TOPO-Blunt PCR cloning vector (Invitrogen) and its sequences were verified (Eurofins MWG Operon) before they were cloned into the *P. falciparum *transfection plasmid pHH1 or pHH2, respectively, to generate pHH1-Δ*gdha *and pHH2-*gdha-GFP*.

### Parasite culture, transfection, determination of IC_50 _values, and parasite growth rate

The *P. falciparum *D10 strain (Papua New Guinea) was cultured according to Trager and Jensen [29] in RPMI 1640 (Invitrogen, UK) containing 11 mM glucose, 0.5% Albumax II (w/v) (Invitrogen, UK), 200 μM hypoxanthine, 20 μg/ml gentamycin (PAA) in human erythrocytes between 0.5% and 5% haematocrit. Parasite cultures were maintained under an atmosphere of reduced oxygen (1% O_2_, 3% CO_2 _and 96% N_2_) and for the growth experiment also at atmospheric oxygen with 5% CO_2 _at 37°C. Parasites were synchronized using sorbitol according to Lambros and Vanderberg [30] and freed from erythrocytes using saponin according to Umlas and Fallon [31]. Parasitaemia was determined using Giemsa stained thin smears and the number of live, isolated parasites was determined using a Neubauer haemocytometer.

Transfection of pHH1-Δ*gdha *and pHH2-*gdha-GFP *into *P. falciparum *erythrocytic stages was performed as described previously Crabb *et al *[32]. WR99210 resistant parasites appeared between 25 and 60 days after transfection. Parasites were cloned by limiting dilution according to Kirkman *et al *[33].

The effects of *N-*methylphenazonium methosulfate, *tert*-butylhydroperoxide and L-cycloserine on the viability of *P. falciparum *erythrocytic stages were determined by measuring the incorporation of [^3^H]-hypoxanthine in the presence of increasing drug concentrations according to Desjardins *et al *[34].

The growth rates of D10 and *gdha *null mutants (D10^Δ*gdha*^) were determined as described by Günther *et al *[35]. Parasites were synchronized twice and after 24 h diluted to 0.5% parasitaemia at 5% haematocrit. Giemsa stained thin smears were prepared daily and parasitaemia determined by counting 1000 erythrocytes per smear. The cultures were maintained at a suitable parasitaemia throughout the 6 day experiment by diluting them 1:5 every second day.

### Fluorescent microscopy

D10 transfected with pHH2-*gdha-GFP*, and resistant to WR99210, at 10% parasitaemia were incubated for 5 min at 37°C with 25 nM MitoTracker Red and 100 μg/ml Hoechst 33342 (both Invitrogen, UK), washed and resuspended in warm complete medium. Cells were viewed with the Applied Precision Deltavision Deconvolution microscope system (Olympus IX-70 invert microscope) fitted with a Coolsnap HQ camera and images were processed with the SoftWoRx software.

### Preparation of genomic DNA, RNA and protein from *P. falciparum*

Parasites were released from erythrocytes by saponin lysis and genomic DNA was isolated using the QIAamp DNA Mini Kit (Qiagen, UK). RNA was extracted from tightly synchronized trophozoites using TRIZOL (Sigma) according to the manufacturer's instructions with modifications as described by Smith *et al *[36]. The final RNA pellet was resuspended in 20-50 μl nuclease free water and stored at -80°C. Protein extracts for western blots were prepared by resuspending the parasite pellets in lysis buffer (PBS, 0.5% (v/v) TritonX-100, 1 mM phenylmethylsulphonyl fluoride, 1 mM benzamidine, 20 μM leupeptin, 10 μM E-64, 2 μM 1,10-phenanthroline, 4 μM pepstatin A) followed by three cycles of freeze/thawing, a 15 min incubation on ice and 5 min centrifugation at 17,000 *g*. For enzyme assays the parasites were lysed as above in 100 mM potassium phosphate buffer pH 7.0 in the presence of protease inhibitors and centrifuged for 30 min at 17,000 *g*. Concentration of soluble protein was determined using the Bradford assay with bovine serum albumin as a standard [37].

### Southern blotting

One to three μg of genomic DNA isolated from D10 and D10 transfected with pHH1-Δ*gdha *were subjected to digestion with NdeI overnight before the DNA was separated on a 0.8% agarose gel and subsequently blotted onto positively charged nylon membrane. After cross-linking the DNA to the membrane, the blot was prehybridized at 55°C before the probe (coding region of the *gdha *gene) was added for an overnight incubation. The probe was prepared using the AlkPhos Direct Labeling and Detection System (VWR, UK) and successive washes were performed according to the manufacturer's instructions. The blot was exposed to autoradiography film (Kodak) for 1 to 16 hours.

### Western blotting

Ten μg of protein from D10, D10^Δ*gdha*^-1 and D10^Δ*gdha*^-2 were separated on 10% or 15% SDS-PAGE gels and blotted onto nitrocellulose using a Transblot Semidry transfer system (BioRad). The membranes were blocked in 3% BSA in PBS (w/v) containing 0.5% Tween 20 (v/v) at 4°C overnight before they were probed with the primary antibodies raised against *P. falciparum *GDHa (1:2,000), *P. falciparum *glutathione *S*-transferase (1:5,000), *P. falciparum *glutathione reductase (1:100,000), *P. falciparum *superoxide dismutase 1 and 2 (1:5,000), *P. falciparum *2Cys-peroxiredoxin 1 (1: 100,000), *P. falciparum *1Cys-peroxiredoxin (1:100,000), *P. falciparum *thioredoxin 1 (1:10,000) and *P. falciparum *isocitrate dehydrogenase (1:30,000). Depending on the respective size of the protein under investigation, an antibody raised against *P. falciparum *branched chain acyltransferase (BCDH-E2 at 1:5,000), detecting a protein of 50 kDa, or the *P. falciparum *1Cys- peroxiredoxin (25 kDa) antibody was used as loading control after it was established that their expression was unchanged in the parent and mutant parasites. The secondary anti-rabbit or anti-rat (for SOD1 and 2 detection) HRP-conjugate antibodies were used at 1:10,000 dilution and the signals were visualized using the Immobilon Western kit (Millipore). Expression relative to the loading control was analysed using LabImage 1D software (Kapelan Bio-Imaging Solutions, Germany). The average expression of the proteins was determined from 2 to 3 independent protein extracts.

### Quantitative real-time PCR

RNA was isolated as described above and treated with TURBO-DNA free (Ambion, UK) before synthesis of cDNA with the RETROscript kit (Ambion, UK) following manufacturer's instructions. Real time PCR reactions were performed in 25 μl using QuantiTect SYBR Green master mix (Qiagen, UK) and primers at a final concentration of 0.3 μM with the 7500 Real Time PCR system (Applied Biosystems, UK). The transcription levels of *gdhb, gdhc, γ-glutamylcysteine synthetase (γgcs), glutathione synthetase (gs) *and *glucose-6 phosphate dehydrogenase (g-6pdh)*, were examined using the primer pairs 5'-GCGTATGCAAAGAGAAGAGGAA-3' and

5'-GTAGAAGTATAGCCGTAGTTGTGTA-3' *(gdhb)*,

5'-TCATCGTCTCCATCATCAGTTC-3' and

5'-CTCACATTACTCTGAGCACATCT *(gdhc)*,

5'-TCCTTGCTCTTACTGCATGTACT-3' and

5'-TTCCGTTCTACAATCAACACTGT-3' *(γgcs)*,

5'-CTTTAGAGCATTATATACACCTAACCA-3' and

5'-CGAACCAACAAGTTGATAAGGTA-3' *(gs)*,

5'-GACCTGAATGATTTTAATTGGAAAGCA-3' and

5'-CATCTATCGGAGTTCGACCTC-3' *(g-6pdh). Seryl-t-RNA synthase *was used as endogenous control, as it had been previously described to have a uniform transcription pattern throughout the parasite life cycle [38,39]. The primer pair

5'-AAGTAGCAGGTCATCGTGGTT-3' and 5'-TTCGGCACATTCTTCCATAA-3' was used and the efficiency of its amplification was similar to *gdhb, gdhc, γgcs, gs *and *g-6pdh*. PCR cycling conditions were 50°C for 2 min, 95°C for 15 min, followed by 40 cycles of 95°C for 15 sec, 54°C for 30 sec and 68°C for 35 sec. Relative expression levels were calculated by the ΔΔCT method (User Bulletin 2, Applied Biosystems, http://www.appliedbiosystems.com).

### GDH activity assays

GDHa activity was detected using a variation of the MTT assay [40]. Five μg of purified recombinant GDHa [3] and 10 μg of parasite extract was loaded onto 10% polyacrylamide gel and resolved under non-denaturing conditions. Gels were washed briefly in 100 mM potassium phosphate buffer pH 8.0 containing 1 mM EDTA and then incubated in the same buffer with 0.3 mg/ml 2-(4-iodophenyl)-3-(4-nitrophenyl)-5-phenyltetrazolium chloride, 0.03 mg/ml phenazine ethosulphate, with 100 mM L-glutamate and 1 mM NADP^+ ^at 37°C, in the dark, until the appearance of dark red coloured bands (formazan deposits). The reaction was stopped by washing gels thoroughly with distilled water.

Total GDH activity was determined in parasite lysate using a spectrophotometric assay based on the oxidation of NAD(P)H or the reduction of NAD(P)^+^, respectively [18]. The forward reaction contained 50 to 100 μg of parasite lysate isolated from D10 or D10^*Δgdha*^-1 or D10^*Δgdha*^-2, 10 mM α-ketoglutarate, 40 mM NH_4_Cl, 200 μM NADPH or NADH in 100 mM potassium phosphate buffer pH 7.0. The decrease in absorbance was followed at 340 nm at 25°C (Uvikon 2501, Shimadzu). The reverse reaction contained 100 μg of parasite extract, 10 mM glutamate, 1 mM NADP^+ ^or NAD^+ ^in 100 mM potassium phosphate buffer pH 8.0. The increase of absorbance was followed at 340 nm at 25°C. The specific activity was determined using the molar extinction coefficient of NAD(P)H of 6,220 M^-1 ^cm^-1 ^at 340 nm.

### Glutathione determination

Glutathione (GSH) levels were determined by HPLC as described by Williams *et al *[41]. Parasites were isolated from 20 ml of tightly synchronized culture (~2 - 5 × 10^7 ^parasites) by saponin lysis, as described above, and parasites were incubated for 45 min at room temperature in 50 μl of 40 mM N-[2-hydroxyethyl]-piperazine-N'[3-propanesulfonic acid], 4 mM diethylenetriamine penta-acetic acid, pH 8.0, containing 0.7 mM tris(2-carboxyethyl)phosphine. 50 μl of 2 mM monobromobimane in ethanol was added and samples heated to 70°C for 3 min before extracts were deproteinized for 30 min on ice by addition of 100 μl 4 M methanesulfonic acid, pH 1.6. Precipitated protein was removed by centrifugation for 40 min at 13,000 *g *at 4°C and the supernatant was analysed by HPLC as described by Williams *et al *[41].

### Metabolic labelling and mass spectrometry

Parasites were synchronized twice, 4 hours apart, and after 35 hours washed in glutamine-free medium and diluted to 7% parasitaemia and 0.7% haematocrit in medium containing 2 mM U-[^13^C] glutamine or U-[^13^C-^15^N] glutamine. At this stage the parasites are early rings and after 32 hours (within 1 cycle) the metabolites from the medium and red blood cells were extracted with 80% methanol on dry ice, as described by Olszewski *et al *[42]. Uninfected red blood cells were incubated in standard culture medium at 5% haematocrit at 37°C for 20 hours prior to the metabolic labelling, as described above, to activate their metabolism. The metabolites were analysed by LC-MS using a Synergy Hydro-RP column (Phenomenex, UK) and an Exactive orbitrap mass spectrometer (Thermo Fisher Scientific, UK) and RAW files were converted and loaded into MAVEN, as detailed by Olszewski *et al *[42].

### Data analysis

Experiments were performed at least in duplicate using independent parasite preparations. Graphs were generated and statistical analyses performed using GraphPad Prism version 5 for Windows (GraphPad Software, San Diego California USA). Significance between the D10^Δ*gdh *^clones and D10 was tested using ANOVA with Newman Keuls post test. The IC_50 _values were calculated by non linear regressing of the sigmoidal dose-response equation (GraphPad Software). Densitometry of protein expression was analysed using LabImage 1D software (Kapelan Bio-Imaging Solutions, Germany).

## Results

### Localization of GDHa in *P. falciparum*

**Figure 1:**
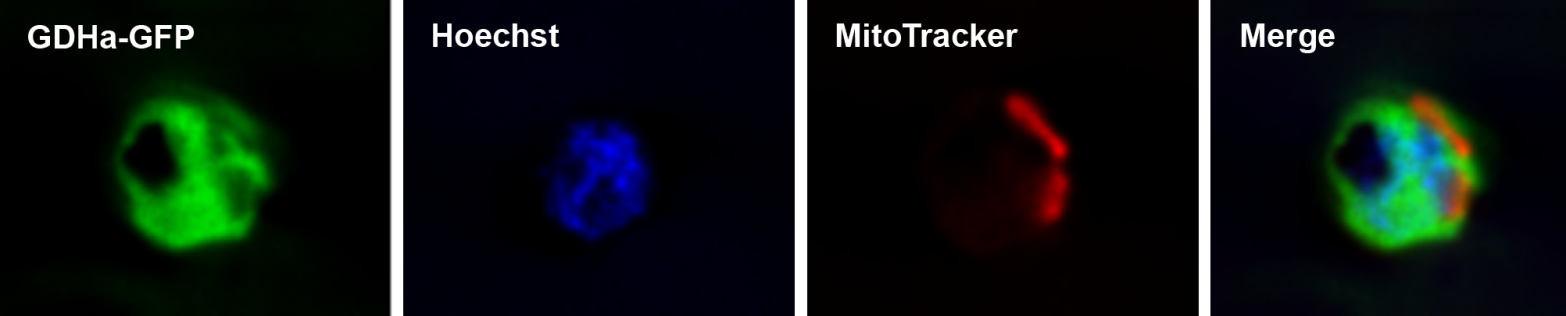
**Cytosolic localization of GDHa **D10 parasites were transfected with pHH1-*gdha-GFP *and WR99210-resistant parasites were analysed by fluorescence microscopy. GDHa-GFP (panel 1) does not co-localize with the nucleus (Hoechst 33342 staining, panel 2) nor with the mitochondrion (MitoTracker staining, panel 3). From the merged image it is clear that GDHa is a cytosolic protein.

The location of GDHa was assessed by expressing the coding region of *gdha*, C-terminally tagged with green fluorescent protein (GFP), from an episome in *P. falciparum *D10 intra-erythrocytic stages. Live parasites were incubated with the nuclear stain Hoechst 33342 and the mitochondrial stain MitoTracker Red and microscopy revealed that the GFP-fusion protein was present exclusively in the cytosol of the parasites (Figure 1).

### Disruption of *gdha *in *P. falciparum*

**Figure 2:**
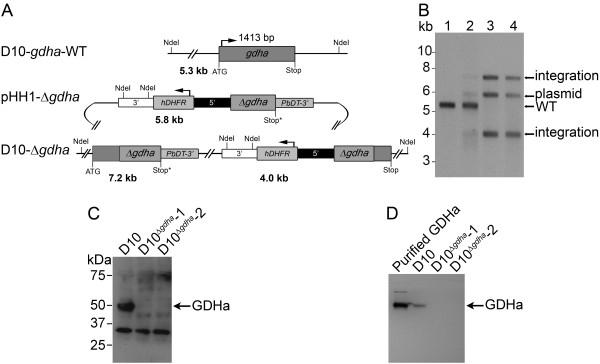
**Generation of *P. falciparum *D10**^**Δ*gdha***^** as confirmed by Southern blot, western blot and enzymatic activity measurement**. (A) Schematic diagram of the endogenous *gdha *gene locus, the pHH1-Δ*gdha *plasmid and the recombined *gdha *locus, following single cross-over recombination between the plasmid and an 841 bp region of *gdha *(D10-Δ*gdha*). The plasmid contains a human dihydrofolate reductase (*hDHFR*) selectable marker under control of the *P. falciparum *calmodulin promoter (5') and flanked by the *P. falciparum *histidine rich protein II 3' UTR (3'), a region homologous to *gdha *(Δ*gdha) *and an artificial 3' UTR (*P. berghei *dihydrofolate reductase/thymidylate synthase 3'UTR, PbDT-3'). NdeI restriction sites and sizes of the resulting diagnostic DNA fragments are indicated (in bold). (B) Southern blot of D10 wild type parasites (lane 1), D10^Δ*gdha *^(lane 2) and two D10^Δ*gdha *^clones (lanes 3 and 4). The 5.3 kb endogenous *gdha *fragment is visible in the wild type and D10^Δ*gdha *^mixed population of parasites before cloning (lanes 1 and 2). In the latter, plasmid (5.8 kb) and the two integration fragments (7.2 kb and 4.0 kb) are also detected. These fragments are still detected in the two clones, D10^Δ*gdha*^-1 and D10^Δ*gdha*^-2 (lanes 3 and 4). (C) Western blot of D10, D10^Δ*gdha*^-1 and D10^Δ*gdha*^-2 protein extracts verifying the absence of GDHa (49 kDa) in D10^Δ*gdha*^-1 and D10^Δ*gdha*^-2. The protein reacting non-specifically with the anti-GDHa antibody (~32 kDa) serves as a loading control. (D) Activity gel of D10, D10^Δ*gdha*^-1 and D10^Δ*gdha*^-2 protein extracts showing GDHa activity in D10 only. Purified recombinant GDHa was included as control.

The D10 strain of *P. falciparum *was used to disrupt the *gdha *gene locus using the plasmid pHH1-Δ*gdha *by single cross-over homologous recombination (Figure 2A). The Southern blot shows that upon diagnostic digest of genomic DNA, isolated from D10, D10^Δ*gdh *^and the clones D10^Δ*gdh*^-1 and D10^Δ*gdh*^-2, the banding pattern obtained with a *gdha*-specific probe is consistent with a disruption of the *gdha *locus by the transfected plasmid pHH1-Δ*gdha *(Figure 2B). The *gdha *band characteristic for the endogenous gene locus (5.3 kb) had disappeared in the cloned parasite lines, while the diagnostic bands for integration of the transfected plasmid into the *gdha *gene locus were present (4.0 kb and 7.2 kb). Additionally, the cloned parasite lines still maintained the transfected plasmid (5.8 kb). D10^Δ*gdh*^-1 and D10^Δ*gdh*^-2 no longer expressed the GDHa protein (49.5 kDa) as shown by western blotting using a GDHa-specific polyclonal antiserum. A non-specific 32 kDa protein is also detected, which serves as a loading control for the parasite extracts and verifies that equal amounts of protein are present in all three lanes of the blot (Figure 2C). The D10^Δ*gdha *^clones also no longer displayed GDHa enzymatic activity as assessed by an in-gel activity assay. Purified recombinant GDHa was used as a control to verify that the reaction band detected in the parasite lysate was the result of GDHa activity (Figure 2D). Clones D10^Δ*gdh*^-1 and D10^Δ*gdh*^-2 were used for further analyses.

Determination of total GDH activity in D10, D10^Δ*gdh*^-1 and D10^Δ*gdh*^-2 showed that the majority of NADPH utilising activity is attributable to GDHa, as this activity is no longer detectable when the GDHa function is lost. The GDH activity of D10 was 36.9 ± 0.35 nmol/min/mg protein while the activity of D10^Δ*gdh*^-1 and D10^Δ*gdh*^-2 was less than 10 nmol/min/mg protein, which was the detection limit of the assay system when 100 μg protein was used. It was only possible to determine the specific activity of the forward reaction for GDH, as the reverse reaction was also below the detection limit at these protein concentrations. The NADH-dependent reduction of α-ketoglutarate also seemed affected in D10^Δ*gdh*^-1 and D10^Δ*gdh*^-2 (10.6 ± 4.1 nmol/min/mg protein and 10.7 ± 4.1 nmol/min/mg protein, respectively) compared to D10 parasites (20.1 ± 0.5 nmol/min/mg protein) suggesting that the NADH-dependent GDH activity is still present in the mutant parasite lines, albeit at lower levels.

### Growth of *P. falciparum *D10^*Δgdha *^and susceptibility to elevated oxidative stress

**Figure 3:**
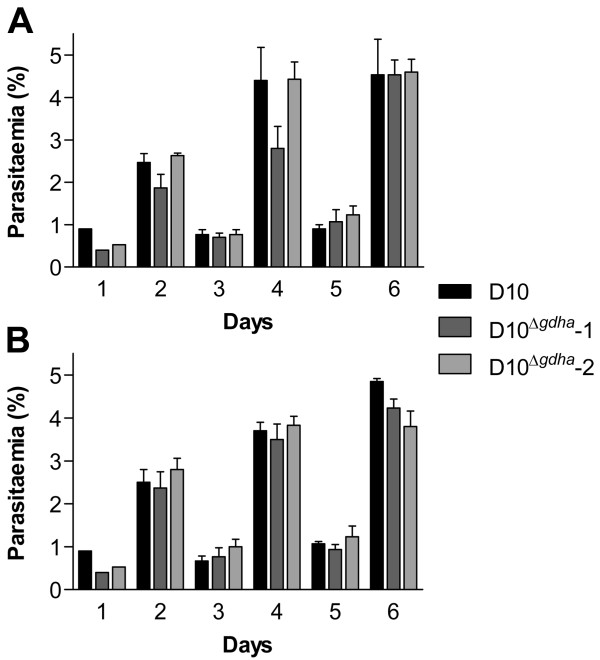
**Growth rate of *P. falciparum *D10 and D10**^**Δ*gdha***^**mutant parasites **Parasites were synchronized and 24 h later diluted to 0.5% parasitaemia (day 1). Thin blood smears were taken daily and the parasite cultures were diluted 1:5 on day 2 and 4. The cultures were maintained at either 1% oxygen (A) or 20% oxygen (B). The experiment was performed in triplicate and the means ± S.D. are shown.

The effect of low (1%) and high oxygen tension (20%) on the growth rate of D10^Δ*gdh*^-1 and D10^Δ*gdh*^-2 was assessed and compared to that of D10 parasites. The absence of GDHa had no effect on the growth of D10^Δ*gdh*^-1 and D10^Δ*gdh*^-2 under low oxygen or elevated oxygen tension (Figure 3). This is surprising because it was previously suggested that GDHa is important for the generation of NADPH, which is required for the parasite's antioxidant defence [13,17]. To test this hypothesis further, it was assessed whether D10^Δ*gdh*^-1 and D10^Δ*gdh*^-2 showed an increased susceptibility towards exogenous or endogenous oxidative stress. However, the IC_50 _values for N-methylphenazonium methosulphate and *tert*-butylhydroperoxide determined for D10^Δ*gdh*^-1 and D10^Δ*gdh*^-2 were similar to those of the D10 wild type parasites (Table 1). These data do not corroborate the hypothesis that GDHa is crucial for a functional antioxidant defence of the parasites.

**Table 1:** IC_50 _values for oxidative stress inducers and an aminotransferase inhibitor for D10 and D10^Δ*gdha *^clones

		**IC**_**50 **_**values (μM)**	
**Parasite line**	**N-methylphenazonium methosulfate**	***Tert*-butylhydroperoxide**	**L-cycloserine**

D10	0.8 ± 0.2	76.7 ± 20.0	145.8 ± 56.6
D10^Δ*gdha*^-1	0.7 ± 0.2	81.8 ± 17.2	113.5 ± 37.5
D10^Δ*gdha*^-2	0.7 ± 0.1	81.3 ± 15.5	111.9 ± 37.4

The effect on parasite viability by the endogenous stressor N-methylphenazonium methosulfate, the exogenous stressor *tert*-butylhydroperoxide and the aminotransferase inhibitor L-cycloserine were determined by the incorporation of [^3^H]-hypoxanthine in the presence of increasing drug concentrations. The IC_50 _values were calculated by non linear regressing of the sigmoidal dose-response equation and shown are the mean ± S.D. of two (N-methylphenazonium methosulfate) or three (*tert*-butylhydroperoxide) independent experiments. For L-cycloserine the mean ± S.E.M of six independent experiments is shown.

### Expression levels of proteins involved in antioxidant reactions

**Figure 4:**
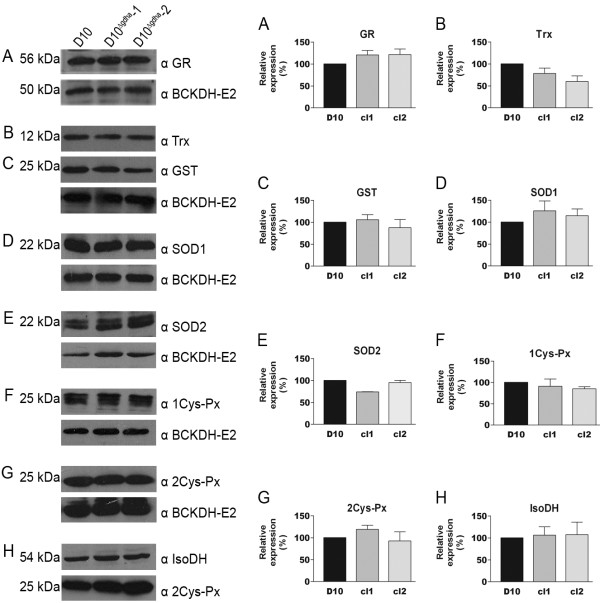
**Western blot analysis of *P. falciparum *proteins involved in antioxidant defence, redox balance or NADP(H) production**. The left panel shows representative western blots of D10, D10^Δ*gdha*^-1 and D10^Δ*gdha*^-2 protein extracts (10 μg) with a range of specific antibodies. The loading control is shown underneath each blot and is either an antibody against the E2 subunit of branched chain α- ketoacid dehydrogenase (50 kDa, panel A - G) or 2Cys- peroxiredoxin (25 kDa, panel H). The two panels on the right show the respective graphs (means ± S.D.) of the densitometry analyses of two or three independent protein extracts. Relative protein levels were calculated by comparing the intensity of the specific protein band with the loading control, with D10 set at 100% (cl1 is D10^Δ*gdha*^-1 and cl2 is D10^Δ*gdha*^-2). A) glutathione reductase (GR, 56 kDa), B) thioredoxin 1 (Trx, 12 kDa), C) blot B re-probed with glutathione S-transferase antiserum (GST, 25 kDa), D) superoxide dismutase 1 (SOD1, 22 kDa), E) superoxide dismutase 2 (SOD2, 22 kDa), F) 1Cys-peroxiredoxin (1Cys-Px, 25 kDa), G) 2Cys-peroxiredoxin (2Cys-Px, 25 kDa), H) isocitrate dehydrogenase (IsoDH, 54 kDa).

In order to assess whether the absence of GDHa led to changes in the expression of proteins involved in antioxidant defences, of which some are NADPH-dependent, western blots of a variety of proteins were performed (Figure 4). The protein levels of the cytosolic 2Cys-peroxiredoxin and 1Cys-peroxiredoxin, the cytosolic and organellar superoxide dismutases 1 and 2, mitochondrial isocitrate dehydrogenase, glutathione reductase and cytosolic thioredoxin 1 did not change significantly in D10^Δ*gdh*^-1 and D10^Δ*gdh*^-2 compared to wild type D10. These results demonstrate that the parasites do not respond to the absence of GDHa by elevating the expression of their antioxidant protein repertoire, which suggests that they are not under enhanced oxidative stress. In addition, the transcript levels of *gdhb, gdhc *and *glucose-6-phosphate dehydrogenase*, the major source of NADPH in the parasite cytoplasm, were analysed by quantitative real time PCR. Similar to the results described above, the mRNA levels of the three genes appeared to be similar in all parasite lines tested. The expression level of *gdhb *is two-fold up-regulated (p < 0.05) in clone D10^Δ*gdh*^-2 but whether this change leads to an increase of GDHb protein remains to be investigated when antibodies against this protein are available (Table 2).

**Table 2:** Quantitative real time PCR of relevant genes from D10 and D10^Δ*gdha *^clones

Fold increase of mRNA compared to D10					
**Parasite line**	***g6pdh***	***gdhb***	***gdhc***	***gs***	***γgcs***

D10^Δ*gdha*^-1	1.8 ± 0.7	1.6 ± 0.2	1.4 ± 0.3	1.4 ± 0.3	2.1 ± 0.2*
D10^Δ*gdha*^-2	2.4 ± 1.0	2.2 ± 0.3*	1.7 ± 0.3	1.5 ± 0.4	2.8 ± 0.2*

The amount of mRNA was calculated using *seryl-t-RNA synthase *as endogenous control and normalized to the amount of mRNA in D10. The values are the mean ± S.D. of two independent experiments. * denotes a significant difference in expression level compared to D10 (p < 0.05).

### Analyses of glutathione levels

Apart from the parasite's enzymatic antioxidant repertoire it is possible that the loss of GDHa function may lead to changes in the level of the non-enzymatic antioxidant and redox buffer glutathione (GSH). The rationale for this suggestion is that the intracellular concentration of GSH is tightly regulated by NADPH-dependent glutathione reductase and decreased levels of NADPH will affect the rate of glutathione disulphide (GSSG) reduction, potentially resulting in a lower amount of GSH and elevated levels of GSSG. The parasites are likely to respond to an increase in GSSG by excreting the tripeptide [43-45] which may result in a decrease in total GSH levels. However, the concentration of total GSH in D10^Δ*gdh*^-1 and D10^Δ*gdh*^-2, compared to D10, was not significantly changed (831 ± 59 nmol/10^10 ^cells; 536 ± 177 nmol/10^10 ^cells and 677 ± 117 nmol/10^10 ^cells in D10, D10^Δ*gdh*^-1 and D10^Δ*gdh*^-2, respectively), suggesting that the reducing power in the parasite was not affected by the absence of GDHa. Concurrently, the mRNA levels of the two genes involved in GSH biosynthesis, *γ-glutamylcysteine synthetase (γgcs) *and *glutathione synthetase *(*gs) *were determined and it was found that *γgcs *mRNA levels were significantly increased between 2.1 and 2.8 fold in the D10^Δ*gdh *^clones compared to D10 (Table 2), but this clearly does not lead to an increase in GSH.

### Metabolic labelling and metabolite analyses

GDHa is thought to be of importance for the conversion of L-glutamate to α-ketoglutarate and therefore instrumental to provide the carbon skeleton for the parasite's TCA pathway intermediates [46]. Synchronized D10, D10^Δ*gdh*^-1 and D10^Δ*gdh*^-2 and non-infected red blood cells were labelled with [U-^13^C] glutamine or [U-^15^N,U-^13^C] glutamine for 32 hours to identify potential changes in the metabolite composition of D10^Δ*gdh*^-1 and D10^Δ*gdh*^-2. Metabolites were extracted and incorporation of heavy isotopes was analysed by liquid chromatography followed by mass spectrometry as previously described [42,47].

The analyses showed that [U-^13^C] glutamine was converted into glutamate and subsequently into α-ketoglutarate, which was then converted into succinate (Table 3). Small amounts of +3 and +4 [^13^C] malate and +3 and +4 [^13^C] fumarate were also detected in the parasites. These results are in agreement with those previously reported by Olszewski *et al *[42]. Surprisingly, the [^13^C] incorporation into α-ketoglutarate and the other TCA pathway intermediates was similar in D10^Δ*gdh*^-1 and D10^Δ*gdh*^-2 compared to D10. These data strongly suggest that GDHa is either not, or not solely, responsible for the conversion of L-glutamate into α-ketoglutarate in the parasites and thus is not essential for providing the carbon skeleton for TCA metabolism. This clearly indicates that this crucial reaction is catalysed by either one of the other two GDH proteins or alternatively by aminotransferases, which are responsible for the transfer of the amino group from glutamate and, depending on the acceptor, results in the production of aspartate, alanine or ornithine [48,49]. This was probed by assessing the effect of L-cycloserine, an inhibitor of aspartate and alanine aminotransferases [50,51]. However, there was no difference in IC_50 _values between D10 wild type and the D10^*Δgdha *^clones suggesting that these aminotransferases do not compensate for the loss of GDHa function (Table 1). The lack of any significant difference in incorporation of [^15^N] into amino acids of wild type and mutant parasites after labelling with U-[^13^C-^15^N]-glutamine further supports this notion (data not shown).

**Table 3:** Metabolic labelling resulting from growth of D10 and D10^Δ*gdha *^clones with U-[^13^C] glutamine-containing medium

**Percentage incorporation of [**^**13**^**C]**			
**Parasite line**	**+5-[**^**13**^**C] glutamate**	**+5-[**^**13**^**C] α-ketoglutarate**	**+4-[**^**13**^**C] succinate**

rbc	60.5 ± 1.1	0	4.9 ± 1.4
D10	48.8 ± 1.8	47.1 ± 3.4	41.6 ± 3.0
D10^Δ*gdha*^-1	45.9 ± 0.8	49.7 ± 4.1	39.9 ± 2.6
D10^Δ*gdha*^-2	45.9 ± 0.6	53.0 ± 3.3	33.4 ± 5.5

The parasite lines were cultured for 32 h with medium containing 2 mM U-[^13^C] glutamine and metabolites extracted and analysed by LC-MS. The percentage of metabolite which has [^13^C] incorporated is shown. The values are the mean ± S.D. of three independent experiments, which were done in triplicate.

## Discussion

The malaria parasite *P. falciparum *contains three genes encoding potential glutamate dehydrogenases; two are NADP(H)-dependent proteins while the cofactor specificity of the third has not been determined to date [2,3]. A previous study, using cross-reacting antibodies, suggested that the parasites possess a cytosolic GDH protein without distinguishing which of the three GDH proteins is detected by the antibodies [52]. It was demonstrated here that GDHa-GFP, expressed episomally in *P. falciparum*, localizes exclusively to the parasite's cytosol while there is no GFP fluorescence present in the mitochondrion or the apicoplast. This is an unusual situation given that in mammals, plants and yeast, GDH are mitochondrial proteins [1,5,53]. However, the localization of GDH can change in senescence in plants, where the protein is distributed in organelle and cytosol [54].

The NAD(H)-dependent plant GDH and the mammalian GDH (using either NAD(H) or NADP(H) as co-factor) are responsible for the oxidative deamination of glutamate to provide α-ketoglutarate as TCA cycle intermediate [1,6,7,53]. The NADP(H)-dependent plant, yeast and bacterial enzymes catalyse the formation of glutamate by assimilating NH_4_^+ ^onto α-ketoglutarate [5,6]. The *Plasmodium *GDHa is a NADP(H)-dependent protein and its kinetic parameters suggest that it can catalyse forward and reverse reactions. It has been argued that the K_m _value for NH_4_^+^, which is between 7 to 10 mM, is high suggesting that the enzyme is more likely to catalyse the formation of α-ketoglutarate and the concomitant generation of NADPH [13]. Consequently it was proposed that the major role for GDHa in *P. falciparum *is to provide NADPH for vital antioxidant reactions and therefore it was deemed a potential target for antimalarial drug design [3,13,14].

In this study, this hypothesis was tested by generating a *P. falciparum *line which no longer contains GDHa protein, by disrupting the *gdha *gene locus. The relative importance of GDHa for overall GDH activity was assessed by determining the enzyme activity in parasite extracts generated from D10 and D10^*Δgdha*^-1 and D10^*Δgdha *^-2. The NADPH-dependent reduction of α-ketoglutarate in D10 was in good agreement with previously reported GDH activities (36.9 nmol/min/mg in D10 versus 63.0 nmol/min/mg, [18]). This NADPH-dependent GDH activity was no longer detectable in the mutant parasite lines, suggesting that GDHa contributes the majority of this GDH activity during intra-erythrocytic parasite development and that the two other proteins, GDHb and GDHc, are only playing a minor role for the NADPH-dependent conversion of α-ketoglutarate to glutamate. The oxidative deamination of glutamate was below detection limit in D10 wild type and mutant parasite lines, suggesting that GDHa is only marginally supportive of this reaction. This is supported by Vander Jagt *et al *[18]. Surprisingly, the loss of GDHa function had no negative effect on the growth of the mutant D10^*Δgdha *^lines under normal culturing conditions. Similarly, applying oxidative stress by either increasing oxygen tension or exposing mutant parasites to pro-oxidants did not result in a growth defect or a change of IC_50 _values of *tert*-butylhydroperxoide and N-methylphenazonium methosulfate. This may be attributable to increased levels of antioxidant proteins in D10^*Δgdha*^, which are up-regulated in response to the loss of GDHa function. However, enzymes that are part of the parasite's antioxidant repertoire did not show any alterations in their expression levels as assessed by western blotting. At mRNA level, no change was detected by real time PCR for the expression levels of *gdhc*, one of the other two genes potentially encoding GDH, or *g-6-pdh*, the enzyme likely to be responsible to provide NADPH for antioxidant reactions. The gene encoding GDHb was found to be up-regulated in clone D10^*Δgdha*^-2, while the other gene that showed a marginal up-regulation of expression in both D10^*Δgdha *^clones was *γgcs*, the rate limiting enzyme of GSH biosynthesis; however no concomitant increase in GSH levels in D10^*Δgdha *^was detectable. Together these data suggest that GDHa has no obvious role in the defence against elevated levels of oxidative stress and that providing NADPH for redox reactions is unlikely to be its major role.

The second reaction product of the oxidative deamination of glutamate by GDHa is α-ketoglutarate, an intermediate of the *P. falciparum *TCA pathway [42,46]. However, the metabolic labelling experiments performed in this study show no difference in the incorporation of the carbon skeleton of [^13^C] labelled glutamine into α-ketoglutarate, succinate and other TCA pathway intermediates between wild type and mutant parasites. This implies that either GDHa is not involved in the oxidative deamination of glutamate or that an alternative pathway compensates for the loss of GDHa function. The latter could be achieved by the two remaining GDH proteins although their mRNA expression levels appear only marginally changed upon *gdha *disruption. However, it is possible that their catalytic activities are elevated in the mutant parasites in response to metabolic changes occurring as a consequence of the absence of GDHa. The activity of GDH, from mammals to bacteria, is highly regulated by the availability of nitrogen and carbon sources [1] suggesting that this may also be possible in the malaria parasites. Clearly the parasites are able to remove toxic ammonia through an efficient aquaglyceroporin suggesting that considerable amounts of the metabolic end-product of amino acid metabolism can be dealt with to avoid intoxication [55].

Another possibility is that aminotransferases replace GDHa function [48,49] but the susceptibility of D10^*Δgdha *^parasites for the inhibitor L-cycloserine, acting against aspartate and alanine aminotransferases [50,51], was not altered compared to wild type parasites. Overall, these data provide evidence that GDHa is not important for the oxidative deamination of glutamate, but may have a role in generating glutamate through NH_4_^+ ^assimilation.

Other organisms with multiple *gdh *genes have a clear distinction between the catalytic activities of NADP(H)- and NAD(H)-dependent enzymes. The NAD(H)- dependent proteins catalyse the generation of α-ketoglutarate while the NADP(H)-dependent GDH enzymes generate glutamate [4-6,8,11]. Glutamate metabolism is highly regulated and depends on the availability of carbon and nitrogen sources. In bacteria it was shown that GDH is only important for nitrogen assimilation when the concentration of NH_4_^+ ^is high and the bacteria are limited for energy (for instance in glucose-limited medium) [56,57]. Similarly, a NADP(H)-dependent GDH is involved in NH_4_^+ ^assimilation in fungi, but only under starvation conditions that limit the reactivity of the glutamine synthetase/glutamate synthase cycle [58,59]. The relatively high K_m _value for NH_4_^+^determined for the parasite GDHa suggests that it may function in a similar way to the bacterial and fungal GDH and synthesizes glutamate for nitrogen storage under starvation conditions, which is supported by the finding that the NADPH-dependent conversion of α-ketoglutarate into glutamate is indeed lost in the mutant D10^*Δgdha *^lines. Under normal growth conditions in bacteria and fungi, glutamate is provided by glutamine synthetase and NADP(H)-dependent glutamine:2-oxoglutarate aminotransferase (glutamate synthase, GOGAT). Genes potentially encoding both of these proteins are present in the *P. falciparum *genome (PFI1110w: potential glutamine synthetase; PF14_0334: potential GOGAT). *P. falciparum *is cultured *in vitro *in the presence of 2 mM glutamine and 11 mM glucose supplying them with plenty of nitrogen and carbon sources. In fact, the supply of high concentrations of glutamine directly provides GOGAT with the substrate it needs to produce glutamate without the necessity to use glutamine synthetase, which depends on ATP hydrolysis. This artificial *in vitro *situation (high glutamine and glucose concentrations) favours the glutamate production via GOGAT so that the parasites do not rely on GDHa activity to synthesize glutamate, explaining the lack of any obvious phenotype of D10^*Δghda *^parasites.

This hypothesis raises important questions with respect to the metabolic functions of GDH in *Plasmodium *and needs further investigation. Clearly the roles of GDHb and GDHc need to be examined concomitantly to fully understand the significance of this interesting metabolic pathway in the parasites. However, these studies exclude a role of GDHa in antioxidant defence and we conclude that GDHa during erythrocytic development is not a suitable drug target given that the protein may only be important for parasite survival under limiting nutritional conditions.

## List of abbreviations

GDHa: glutamate dehydrogenase a; GDHb: glutamate dehydrogenase b; GDHc: glutamate dehydrogenase c; GSH: glutathione; GSSG: glutathione disulphide; RBC: red blood cell; TCA: tricarboxylic acid.

## Competing interests

The authors declare that they have no competing interests.

## Authors' contributions

JS carried out the molecular genetic studies, performed most of the phenotype analyses of *gdha *null mutant parasites including the metabolic labelling of parasites and analyses of metabolite datasets. In addition she made significant contributions to study design, data analyses and interpretation and also wrote part of the manuscript; JP carried out the western blot analyses and participated in other parts of the phenotype analyses of *gdha *null mutant parasites; IA cloned the knockout and GFP constructs and performed the in-gel GDHa assays; EMP determined the GSH level of wild type and mutant parasites; KO determined the metabolites after metabolic labelling and assisted in data analyses; ML contributed to data analyses and provided critical input into drafting the manuscript; PCE made substantive intellectual contributions to this study; SM made substantive intellectual contributions to study design, data analyses, interpretation and acquisition of data and wrote the manuscript. All authors have read and approved the final manuscript.
